# Sulfation and Enhanced Antioxidant Capacity of an Exopolysaccharide Produced by the Medicinal Fungus *Cordyceps sinensis*

**DOI:** 10.3390/molecules18010167

**Published:** 2012-12-24

**Authors:** Jing-Kun Yan, Wen-Qiang Wang, Hai-Le Ma, Jian-Yong Wu

**Affiliations:** 1 School of Food and Biological Engineering, Jiangsu University, Zhenjiang 212013, Jiangsu, China; E-Mails: jkyan_27@163.com (J.-K.Y.); mhl@ujs.edu.cn (H.-L.M.); 2 Department of Applied Biology & Chemical Technology, The Hong Kong Polytechnic University, Hung Hom, Kowloon, Hong Kong; E-Mail: wwq19790511@tom.com (W.Q.-W.); 3 Physical Processing of Agricultural Products Key Lab of Jiangsu Province, Zhenjiang, 212013, Jiangsu, China

**Keywords:** *Cordyceps sinensis*, exopolysaccharide, sulfation, molecular weight, antioxidant activity

## Abstract

EPS-1 was an exopolysaccharide produced by the medicinal fungus *Cordyceps sinensis* (Cs-HK1). In the present study, EPS-1 was sulfated with chlorosulfonic acid (CSA)-pyridine (Pyr) at different volume ratios, yielding four sulfated derivatives, SEPS-1A, B, C and D, with different degrees of substitution (DS: 0.25–1.38) and molecular weights (17.1–4.1 kDa). The sulfation of EPS-1 occurred most frequently at the C-6 hydroxyl groups due to their higher reactivity. In aqueous solution, the native EPS-1 formed random coils or aggregated networks, but the sulfated derivatives formed single helices. The antioxidant activities of the sulfated EPS-1 derivatives for scavenging hydroxyl radicals (•OH) and 2,2-azinobis-3-ehtylbenzothiazolin-6-sulfonic acid radicals (ABTS•+) were significantly increased with increasing DS and decreasing molecular weight (MW). Sulfation has thus been shown to be an effective and favorable strategy for improving the physico-chemical properties and bioactivities of fungal polysaccharides.

## 1. Introduction

Reactive oxygen species (ROS), including free radicals and peroxides, are regarded as the culprits of numerous human diseases such as carcinogenesis, atherosclerosis, Alzheimer’s disease, aging and degenerative processes [1]. Because of their high reactivity, the excess ROS in the human body can cause the destruction of biomolecules, DNA strand breakage, protein denaturation, enzyme inactivation, and polysaccharide degradation [1,2]. Antioxidants are substances that can eliminate ROS, thus preventing or delaying the oxidation of cellular components. Natural products provide the most diverse and abundant sources of antioxidants for health protection and disease prevention. Currently the most common and useful natural antioxidants include polyphenols, flavonoids, saponins, tannins, alkaloids and polysaccharides [3]. Polysaccharides from plants and fungi represent one of the most promising classes of antioxidants for preventing oxidative damage in foods and living organisms due to their wide availability and low toxicity [4]. Many recent studies have demonstrated that sulfated polysaccharides (SPS) isolated from marine algae and other sources have remarkable bioactivities, such as anticoagulant and antithrombotic [5], antitumor [6], antiviral [7], and antioxidant properties [8]. Their activities are closely related to the presence of polyanionic charges. Therefore, sulfation has been widely applied to modify neutral polysaccharides [9] and to improve water solubility, and bioactivities [10]. Sulfation of polysaccharides is a useful approach to attaining new pharmacological agents with possible therapeutic uses, and to develop new health products or cosmetics.

Cordyceps (*Cordyceps sinensis*) is a valuable medicinal fungus and polysaccharides (PS) are its major bioactive constituents, with reported anticancer, immunomodulation and antioxidant activities [11,12]. Cs-HK1 is a Cordyceps fungus and has been applied to produce exopolysaccharides (EPS) in mycelial culture [13]. The crude EPS isolated from the Cs-HK1 culture broth showed moderate antioxidant and radical scavenging activities [14]. So far, no studies have been performed to improve the antioxidant activity by sulfating the PS molecules from natural or cultured Cordyceps fungal species. In the present work, a PS molecule EPS-1 purified from the crude EPS produced by the Cs-HK1 fungus was subjected to sulfation, yielding sulfated derivatives with different degrees of substitution (DS). Their molecular properties and antioxidant activities were evaluated and the mechanism of their antioxidant action was discussed.

## 2. Results and Discussion

### 2.1. The Sulfation of EPS-1

**Figure 1:**
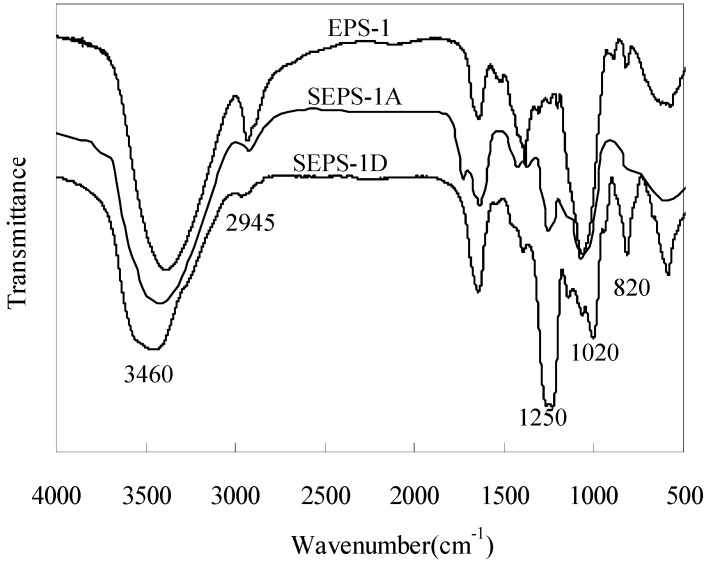
FT-IR spectra of EPS-1 and sulfated derivatives, SEPS-1A and SEPS-1D.

The crude exopolysaccharide (EPS) was isolated from the liquid medium of Cs-HK1 mycelial culture by ethanol precipitation, and purified through deproteinization, decolorization and dialysis [Molecular Weight Cut Off (MWCO) 12–14 kDa], yielding the EPS-1 fraction. The results of ultraviolet (UV) spectrum (data not shown) and Fourier transformed infrared spectrum (FT-IR) analysis (Figure 1) indicated that EPS-1 was a neutral polysaccharide free of monosaccharides, uronic acids, proteins and nucleic acids, and was sufficiently pure for the sulfation experiment.

Table 1 shows the major molecular parameters of four sulfated EPS-1 derivatives obtained at four CSA/Pyr volume ratios. The degree of substitution (DS) of sulfated derivatives increased almost linearly with CSA/Pyr ratio and the yield of SEPS-1 fractions also increased steadily with the CSA/Pyr ratio. SEPS-1D had the highest DS of 1.38 and yield of 247 mg. This was in accord with the previous work that the higher molar ratio of sulfating reagent resulted in a higher DS under constant reaction conditions [10].

**Table 1:** Yields and characteristics of sulfated EPS-1 derivatives.

EPS fraction	CSA:Pyr(v/v)	Yield (mg) ^a^	DS	M_w_^b^ (kDa)	M_n_^b^ (kDa)	P ^b^ (M_w_/ M_n_)
EPS-1	—	—	—	38.0	25.9	1.47
SEPS-1A	1:8	197	0.25	17.1	16.4	1.04
SEPS-1B	1:4	216	0.46	13.2	12.1	1.09
SEPS-1C	1:2	229	0.93	9.4	7.8	1.20
SEPS-1D	1:1	247	1.38	4.1	3.9	1.05

^a^ Starting with 200 mg EPS-1. ^b^ M_w_: Weight-average molecular weight; M_n_: Number-average molecular weight; P: Polydispersity index.

### 2.2. IR Spectra of EPS-1 and SEPS-1

As seen from the IR spectra (Figure 1), two characteristic absorption bands, which were absent in the spectrum of EPS-1, appeared in the spectra of SEPS-1A and SEPS-1D, one near 1250 cm^−1^ and the other near 820 cm^−1^. The former was attributed to the presence of an asymmetrical S=O stretching vibration and the latter to a symmetrical C-O-S group associated with the C-O-SO_3_ group. Both peaks showed an increase in the intensity with DS from SEPS-1A to -1D. Moreover, the peak at 2940 cm^−1^ was attributed to C-H and that at 1020 cm^−1^ to C-O-H, both becoming weaker with increasing DS. These IR characteristics confirmed that the sulfation reaction of EPS-1 had been accomplished [15]. It was also noticeable that SEPS-1D had a peak at 820 cm^−1^, indicating sulfation at the C-6 of galactose in EPS-1. In general, the C-6 hydroxyl group of polysaccharides has a higher reactivity for sulfation than the C-2 and C-3 hydroxyl groups [16].

### 2.3. Changes in Molecular Weight and Chain Conformation

As shown in Table 1, the MW of EPS-1 was significantly reduced by sulfation and decreased steadily with the increase of CSA/Pyr ratio, indicating the degradation of polysaccharide chain during the sulfation reaction. The degradation of polysaccharides during the sulfation can be mainly attributed to the acid hydrolysis by CSA during the sulfation process [17]. In addition to the MW level, the polydispersity index was also reduced with the sulfation, indicating of a narrower or more uniform molecular weight distribution (MWD). The –SO_3_H groups in the derivative enhance the steric hindrance between the polymer chains, leading to more ordered and expanded conformation of the sulfated derivatives [18], improving the homogeneity of the sulfated derivatives in aqueous solutions.

**Figure 2:**
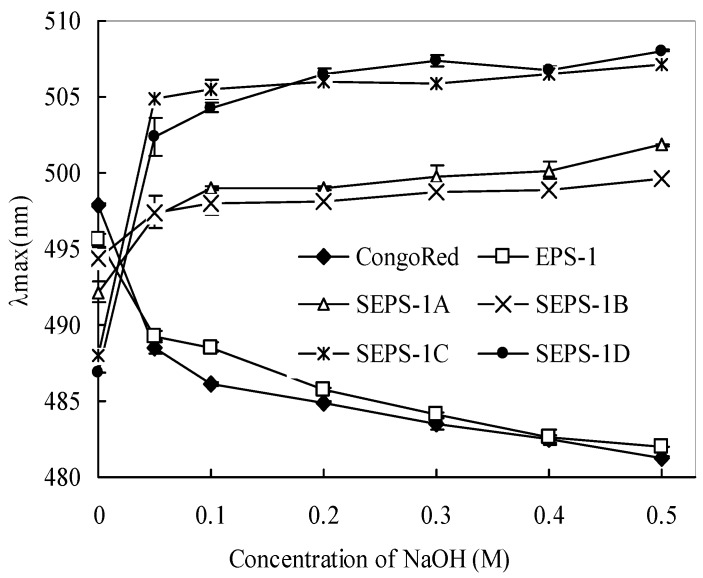
Change in the absorption maximum (λ_max_) of Congo red and Congo red-EPS-1 complex solutions at various NaOH concentrations.

Figure 2 shows the change of maximum absorbance (λ_max_) of polysaccharide-Congo red solutions with alkaline concentrations from 0 to 0.5 M. In the presence of NaOH, the EPS-1-Congo red solution exhibited a blue shift (or λ_max_ drop) from ~495 nm to 485 nm (by 10 nm at 0–0.20 M NaOH), indicating that EPS-1 formed random coils or large aggregates in the aqueous solution. However, the sulfated derivatives with Congo red solutions exhibited a notable red shift (or λ_max_ increase) from 0–0.2 M NaOH. The red shift with the sulfated EPS-1 derivatives in the Congo red solution suggests the presence of helices in solution. Moreover, with the increase in DS, the red shift of λ_max_ from ~6 nm (from ~493 nm to 499 nm for SEPS-1A) increased to 20 nm (from ~486 nm to 506 nm for SEPS-1D) at 0.20 M NaOH with Congo red solution, suggesting the formation of single helices by the sulfated derivatives with a higher DS. The change in chain conformation with the sulfation further indicates that the introduction of sulfate groups in EPS-1 increased the steric hindrance between the polymer chains, leading to the disruption of aggregates by sulfation to form single helices for interaction with Congo red [19]. In addition, the relative stable λ_max_ of SEPS-1-Congo red complexes at 0.2 M or higher NaOH concentrations suggests that the stabilization of the higher order SEPS-1 conformations at a higher alkaline concentration. 

### 2.4. Antioxidant Activities of Sulfated EPS-1 Derviatives

**Figure 3:**
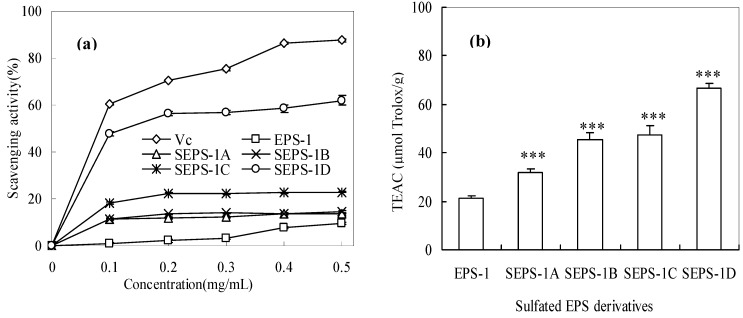
Antioxidant activity of EPS-1 and its sulfated derivatives measured: (**a**) hydroxyl radical scavenging activity; (**b**) Trolox equivalent antioxidant capacity. (***: significant effect at *p* < 0.01; EPS-1 significant effect at *p* < 0.05).

Figure 3 shows the results of the antioxidant activity assays of the EPS-1 fractions. Both antioxidant assays showed that the sulfated EPS-1 derivatives had higher activities than the native EPS-1, and the activities were higher with the increase in DS.

Figure 3a shows the concentration-dependent hydroxyl radical-scavenging activities of four sulfated EPS-1 derivatives from 0.1–0.5 mg/mL. SEPS-1D with the highest DS had the highest activity with an EC_50_ value about 0.13 mg/mL, which was close in magnitude to that of ascorbic acid (Vc), 0.08 mg/mL. The results suggested that the sulfate groups play an important role in the scavenging of hydroxyl radicals. Wang *et al*. [20] also measured the hydroxyl radical-scavenging activity of a sulfated polysaccharide from *Artemisia sphaerocephala* SASP-3 and obtained an EC_50_ of 1.52 mg/mL. In the comparison of the EC_50_ values, the SEPS-1D derived from EPS-1 had a much stronger hydroxyl radical scavenging activity than SASP. SEPS-1C with a similar DS to SEPS-1D had a lower hydroxyl radical scavenging activity than SEPS-1D, which was probably due to the higher molecular weight. As suggested by Yang *et al*. [21], polysaccharides with higher sulfate content and lower molecular weight usually have stronger scavenging effects on hydroxyl radicals. Our previous study also showed that lower MW fractions (~3,000 Da) of EPS-1 derived from acidic hydrolysis possessed stronger hydroxyl radical scavenging activity [22].

Figure 3b shows that the ABTS•+ scavenging activity of EPS-1 and its sulfated derivatives, with the TEAC value increasing from the lowest 21.0 (*p* < 0.05) for EPS-1 to the highest 66.5 μmol Trolox/g (*p* < 0.01) for SEPS-1D. In comparison of the TEAC values, SEPS-1D also had a higher activity than an acid hydrolysed EPS-1 fraction with a MW of ~3 kDa having a TEAC value of 59 μmol Trolox/g [22]. The comparison suggests that the substitution of hydroxyl groups by sulfate groups (–OSO_3_H) in the EPS-1 molecule offers stronger radical scavenging activity.

**Scheme 1:**
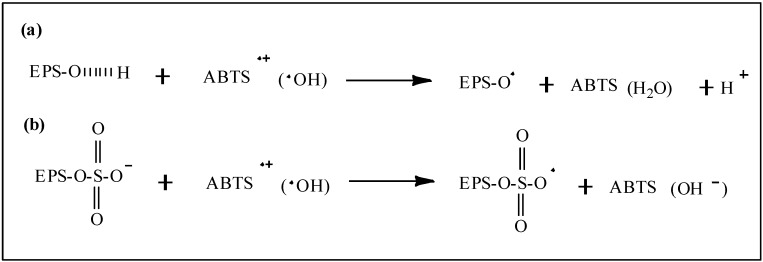
Proposed mechanisms for the radical scavenging action of the EPS-1 and derivatives: (**a**) H-atom transfer (HAT) between EPS-1 and free radicals; (**b**) electron transfer (ET) between sulfated EPS-1 molecules and free radicals.

Scheme 1 presents the proposed mechanisms for the radical scavenging actions of EPS-1 and its sulfated derivatives, the hydroxyl groups of EPS-1 react with •OH through the typical hydrogen-abstraction reaction while the sulfated EPS-1 molecule donates an electron from the long-pair electron of the sulfate group to form a stable free-radical ion. As higher electron-cloud density can offer stronger electron-donating capacity, the sulfate groups increase the electron-cloud density of polysaccharides to enhance the radical scavenging capacity. Moreover, the MW of sulfated polysaccharides is a more important factor for scavenging free radicals than DS by ET reactions. 

It has been established that natural antioxidants such as phenols (ArOH) scavenge free oxygen-centered radicals via two major mechanisms, hydrogen atom transfer (HAT) reactions and electron transfer (ET) reactions [23,24]. Recently, Kishk and Al-Sayed reported that the hydroxyl radical scavenging mechanism of polysaccharides was perhaps similar to that of phenol compounds by HAT reactions [25]. Tsiapali *et al*. also reported that the hydroxyl radical scavenging of polysaccharides may be due to the anomeric hydrogen from one of the internal glucose units reacting with •OH by the HAT mechanism [26]. However, the HAT reaction is more likely to occur in neutral polysaccharides, while the ET mechanism is likely to occur in the acidic polysaccharides (with modified or contained uronic acid) as confirmed by Chen *et al*. [27]. ABTS•+ can also accept an electron or hydrogen radical to become a relatively stable diamagnetic molecule. The scavenging capacity of the sulfated polysaccharide derivatives on ABTS•+ can be attributed to the negative charge of the sulfate groups through the ET reaction mechanism (Scheme 1b).

## 3. Experimental

### 3.1. Materials and Chemicals

The EPS-1 polysaccharide fraction was purified from the crude EPS produced by mycelial culture of the Cs-HK1 fungus in a liquid medium as reported previously [22]. ABTS (2,2-azinobis-3-ehtylbenzothiazolin-6-sulfonic acid) and Trolox (6-hydroxy-2,5,7,8-tetramethylchroman-2-carboxylic acid) were purchased from Calbiochem/EMD (Gibbstown, NJ, USA). Hydrogen peroxide (H_2_O_2_), brilliant green and Congo red dye were from Sigma-Aldrich Chemical Co. (St. Louis, MO, USA). Other chemical reagents were of analytical grade obtained from authorized suppliers. All aqueous media and reagent solutions were made with de-ionized water.

### 3.2. Sulfation of EPS-1

**Scheme 2:**
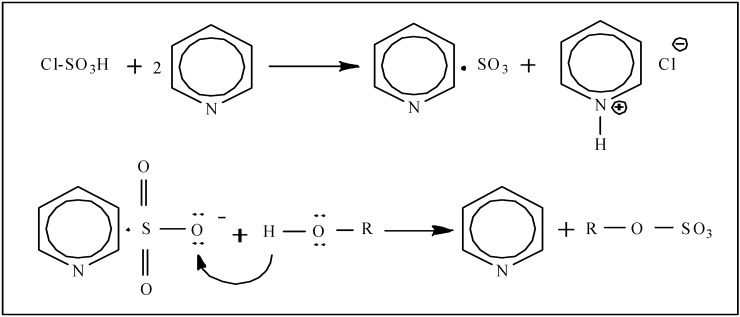
Reaction mechanism for sulfation of EPS (R) by chlorosulfonic acid-pyridine.

Sulfation of EPS-1 was carried out by using chlorosulfonic acid (CSA)-pyridine (Pyr) as the sulfating reagent by the procedure described in [20]. The EPS-1 solid (200 mg) was dispersed in anhydrous formanide (20 mL); the sulfating reagent (20 mL) at a chosen CSA/Pyr (v/v) was added dropwise to the EPS-1 dispersion. The reaction mixture was maintained at 60 °C for 3 h with constant stirring, and then cooled to room temperature and neutralized with 2 M NaOH solution, followed by ethanol precipitation. The precipitate was collected and redissolved in deionized water, and dialyzed (MWCO 3,500 Da) against slightly alkaline water (pH 9) to remove pyridine for 24 h, and then dialyzed against deionized water for 48 h. The solution was evaporated and freeze-dried to give the final sulfated EPS-1 derivative. Four derivatives were attained with four different CSA/Pyr volume ratios, 1:8, 1:4, 1:2, and 1:1, designated respectively as SEPS-1A, B, C and D. The derivatives were stored in a desiccator at room temperature before use. The reaction mechanism for the EPS-1 sulfation by CSA-Pyr reagent is shown in Scheme 2 [28].

The sulfur content of SEPS-1 fractions was determined as reported previously [29] using sodium sulfate as a standard, and the degree of substitution (DS) was calculated by DS = 162 × SO_4_^2−^%/100 − (96/98 × SO_4_^2−^%).

### 3.3. Analysis of EPS-1 and SEPS-1 Molecular Properties

Molecular weight and distribution (MWD) of SEPS-1 was determined by high pressure gel permeation chromatography (HPGPC) with the instruments and conditions as described in detail by Yan *et al*. [22]. The HPGPC was calibrated with 10 dextrans MW standards from 5–1,400 kDa (Sigma, St. Louis, MO, USA), and the data was analyzed with Breeze V3.3 software (Waters Corporation, Milford, MA, USA). Infrared (IR) spectra of EPS-1 and SEPS-1 fractions were recorded in the 4,000–500 cm^−1^ wave number region on a Vector 33 FTIR instrument (Bruker Co., Karlsruhe, Germany).

Congo red test was performed of EPS-1 and SEPS-1 to examine the chain conformations [30]. The EPS-1 fractions were dissolved at 1 mg/mL in water containing 91 μM Congo red and then treated with NaOH at various concentrations (0–0.5 M) and the visible light absorption spectrum was recorded from 400 nm to 700 nm at room temperature with a spectrometer against de-ionized water as the blank.

### 3.4. Antioxidant Activity Assays

Two assays were applied to measure the antioxidant activities of EPS-1 fractions, hydroxyl radical scavenging assay and Trolox equivalent antioxidant capacity (TEAC) assay.

The hydroxyl radical scavenging activity was determined based on the Fenton reaction as described by He *et al*. [31]. The Fenton reaction solution was freshly prepared by mixing 0.435 mM brilliant green (1.0 mL), 0.5 mM FeSO_4_ (2.0 mL) and H_2_O_2_ (1.5 mL, 3.0% v/v). The EPS-1 sample was added to the reaction solution and incubated at room temperature for 20 min, followed by absorbance measurement at 624 nm. The hydroxyl radical scavenging activity (%) was given by [(A − A_o_)]/[(A_b_ − A_o_)] × 100, where A, A_o_ and A_b_ were the absorbance values of the reaction solution in the presence of the EPS-1 sample, the control (reaction solution), and the blank (water). Vitamin C (V_c_) was used as a positive antioxidant reference.

The TEAC assay of the EPS-1 fractions was performed as reported before [14]. The ABTS•+ radical solution was freshly prepared by the reaction of K_2_S_2_O_8_ (13.2 mg) with 7.4 mM ABTS solution (20 mL) for about 12–16 h at room temperature in the dark, yielding a blue-green solution. The solution was diluted with PBS (pH 7.4) to attain an absorbance value of 0.7 at 734 nm for the assay. The EPS-1 samples and Trolox were predissovled in PBS (pH 7.4) and 0.1 mL of each sample solution was mixed with 3.9 mL of the diluted ABTS·+ for 20 min at room temperature. The absorbance was measured at 734 nm and the radical-scavenging activity (%) was given by (1 − A/A_0_) × 100, where A and A_0_ were the absorbance values of ABTS•+ solution in the presence and absence of the test samples, respectively. The TEAC values were derived from the calibration curve obtained by Trolox in the concentration range of 0–30 µM.

### 3.5. Statistical Analysis

All treatments and activity assays were performed in triplicates and the results were represented by their mean ± standard deviation (SD). Data were analyzed by a one-way analysis of variance (ANOVA) and processed with Prism 5.0. Student’s *t*-test was used to evaluate significance of any differences between groups.

## 4. Conclusions

Four sulfated derivatives with different degrees of substitution and molecular weights were synthesized from EPS-1 isolated from the cultured broth of *Cordyceps sinensis* (Cs-HK1) by the chlorosulfonic acid-pyridine method. The sulfation resulted in a significant reduction of molecular weight and changes in chain conformation. The most promising result from the sulfation was a significant improvement of the antioxidant (radical scavenging) activities of the EPS-1. Overall the present study has shown that sulfation is a simple and effective approach for modification of the molecular properties and improvement of the bioactive functions of natural polysaccharides. There is still a need to optimize the sulfation process and reaction conditions for the production of homogenous polysaccharide structures in high yields and with desired properties and bioactivities.
